# Tongue image analysis for accurate prediction of nutritional risk screening in cancer patients during radiochemotherapy: a feature selection network and aliasing attention mechanism approach

**DOI:** 10.3389/fnut.2025.1752250

**Published:** 2026-01-12

**Authors:** Bowen Yang, Aohan Li, Abdul Haleem Mohib, Xu Qiao, HuaiDong Li, Cong Wang, Chang Liu, Henan Zhang, Yukun Zhang, Shuying Li, Shanghui Guan, Shasha Zhao

**Affiliations:** 1 Center of Intelligent Medicine, School of Control Science and Engineering, Shandong University, Jinan, Shandong, China; 2 Department of Radiation Oncology, Shanghai Pulmonary Hospital, Tongji University School of Medicine, Shanghai, China; 3 Department of Hospitality and Business Management, Technological and Higher Education Institute of Hong Kong, Hong Kong, China; 4 Department of Radiation Oncology, Qilu Hospital, Shandong University, Jinan, Shandong, China; 5 Department of Nutrition, Qilu Hospital, Shandong University, Jinan, Shandong, China

**Keywords:** attention mechanism, computer aided diagnosis, NRS2002, nutriology, tongue image diagnosis, tumornutrition analysis

## Abstract

**Background:**

The Nutritional Risk Screening 2002 (NRS2002) is a widely adopted tool for assessing nutritional risk in patients. This study introduces a novel, non-invasive, efficient, and accurate screening approach to complement the traditional NRS2002 assessment, addressing its inherent limitations.

**Methods:**

A dataset comprising 672 tongue images from 470 tumor patients was collected. A new predictive analysis system for NRS2002 was developed by integrating two model branches. Machine learning was employed for risk prediction, and a ResNet50 neural network was utilized to extract high-dimensional features from tongue images. This architecture was enhanced with Shuttle Attention mechanism. The final predictions were derived through the fusion of both model branches.

**Results:**

The fusion of the two branches significantly improved the model’s ability to capture complex features. For both at-risk and risk-free cohorts, the system demonstrated optimal classification performance across three key metrics: AUC = 0.919, ACC = 0.927, and Recall = 0.888. In ablation studies, the SelectNet module improved ACC and AUC by 17.25 and 16.14%, respectively. Furthermore, the integration of the shuttle attention mechanism led to additional gains, with AUC and ACC increasing by 3.89 and 1.94%, respectively.

**Conclusion:**

We successfully developed and validated an NRS2002 nutritional risk prediction model based on tongue image characteristics. This tool has the potential to minimize human error, improve dynamic performance, and provide non-invasive, accurate nutritional risk screening. It represents a step forward in personalized medicine and holds substantial clinical value.

## 1 Introduction

Adequate and balanced nutrition supports physiological homeostasis and immune competence, whereas nutritional deficiencies or excesses can disrupt metabolic regulation and increase disease susceptibility (1). Nutritional risk screening is directly related to clinical prognosis and medical reimbursement policies, and it is widely used in hospitals. The Nutritional Risk Screening 2002 (NRS2002) scheme is globally recognized for inpatient nutritional risk assessment. Although NRS2002 (≥3 points) is a key criterion for initiating enteral/parenteral nutrition therapy, its clinical application involves limitations such as complicated operation and bias in dietary review, requiring nursery-led weight monitoring and dietary review. Moreover, it requires a median time of 5 min for a single screening, which affects dynamic clinical monitoring of patients’ nutritional risk. These limitations highlight the urgent need for non-invasive, precise, and efficient alternative tools to optimize nutritional risk across different clinical populations.

Traditional Chinese medicine (TCM) (2) is a medical, scientific, and cultural heritage that has been empirically applied and preserved in China for thousands of years (3, 4). Tongue diagnosis is one of the most important aspects of TCM diagnosis (5). According to TCM theory, tongue features can reflect human health as a whole, and changes in the color, shape, and texture of the tongue provide valuable pathological and physiological information. Previous studies have shown that tongue features are closely related to gastric cancer, colon cancer, and other digestive diseases, and that nutritional status can be quickly and accurately reflected in tongue images. Thus, tongue image features may be used to quickly predict nutritional risk using NRS2002. However, traditional diagnostic methods rely heavily on the personal experience and subjective judgment of clinicians, leading to limited repeatability. In contrast, emerging artificial intelligence (AI) and machine learning technologies offer automated analysis with high sensitivity, which is crucial for precisely capturing subtle pathological information from lesion images (6). Such advancements in imaging and diagnostic technologies not only minimize human error but can also ultimately contribute to the formulation of personalized treatment plans (7).

Artificial intelligence (AI) can be used to screen, diagnose (8), and treat a variety of diseases, especially those of the digestive system, by accurately analyzing diagnostic clinical images, identifying therapeutic targets, and processing large datasets. The application of AI in TCM tongue-image diagnosis focuses on the standardization of tongue feature extraction to eliminate differences caused by manual interpretation (9).

This paper presents a non-invasive NRS2002 nutritional risk dynamic prediction model based on tongue image features to address the limitations of traditional assessment methods by leveraging deep learning. Considering the problems of multi-modal data dependence and lack of dynamic monitoring capability and interpretability in existing machine learning methods, an innovative dual-modal feature fusion framework was designed.

## 2 Materials and methods

### 2.1 Overview framework

**Figure 1:**
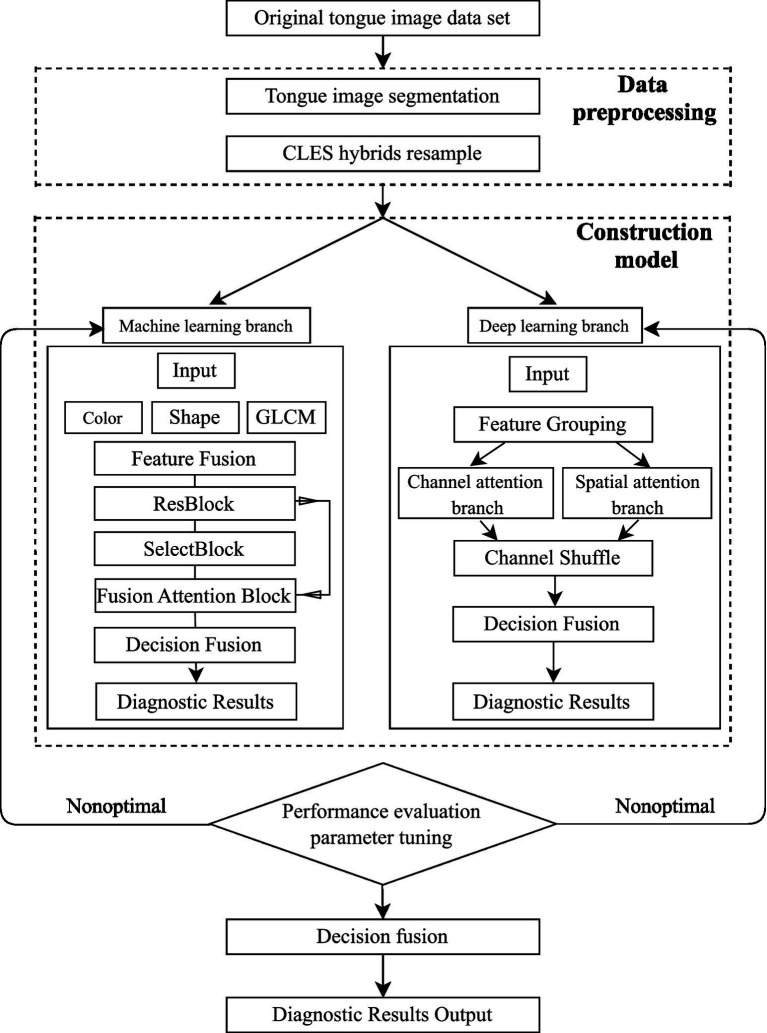
Flowchart illustrating the construction of the NRS2002 classification and prediction dual-channel information fusion model.

The overall structure is shown in Figure 1, which involves image classification based on feature selection and a classification model combining depth spatial information and regional location information of the attention mechanisms. Finally, the score of the prediction results of the two branches was considered as the feature, and a logistic regression model was constructed to fuse the two to obtain the final prediction result.

Low-dimensional manual features were extracted based on the tongue diagnosis of TCM theory, the tongue image quantification index system was optimized through a self-designed feature selection network, higher-order semantic features were extracted and combined with an improved deep residual network, and an inter-dimensional attention mechanism was introduced to analyze the spatial topological relationship of tongue images (such as regional tongue coating thickness changes). In addition, a mixed sampling strategy was constructed to solve the data imbalance problem. The framework can enable end-to-end risk assessment of pure tongue image inputs, significantly reducing the screening time, with high precision and strong interpretability. Moreover, this scheme is expected to serve as a new objective evaluation tool and efficient clinical solution for the dynamic monitoring of nutritional risk in cancer patients during treatment.

### 2.2 Research data

**Figure 2:**
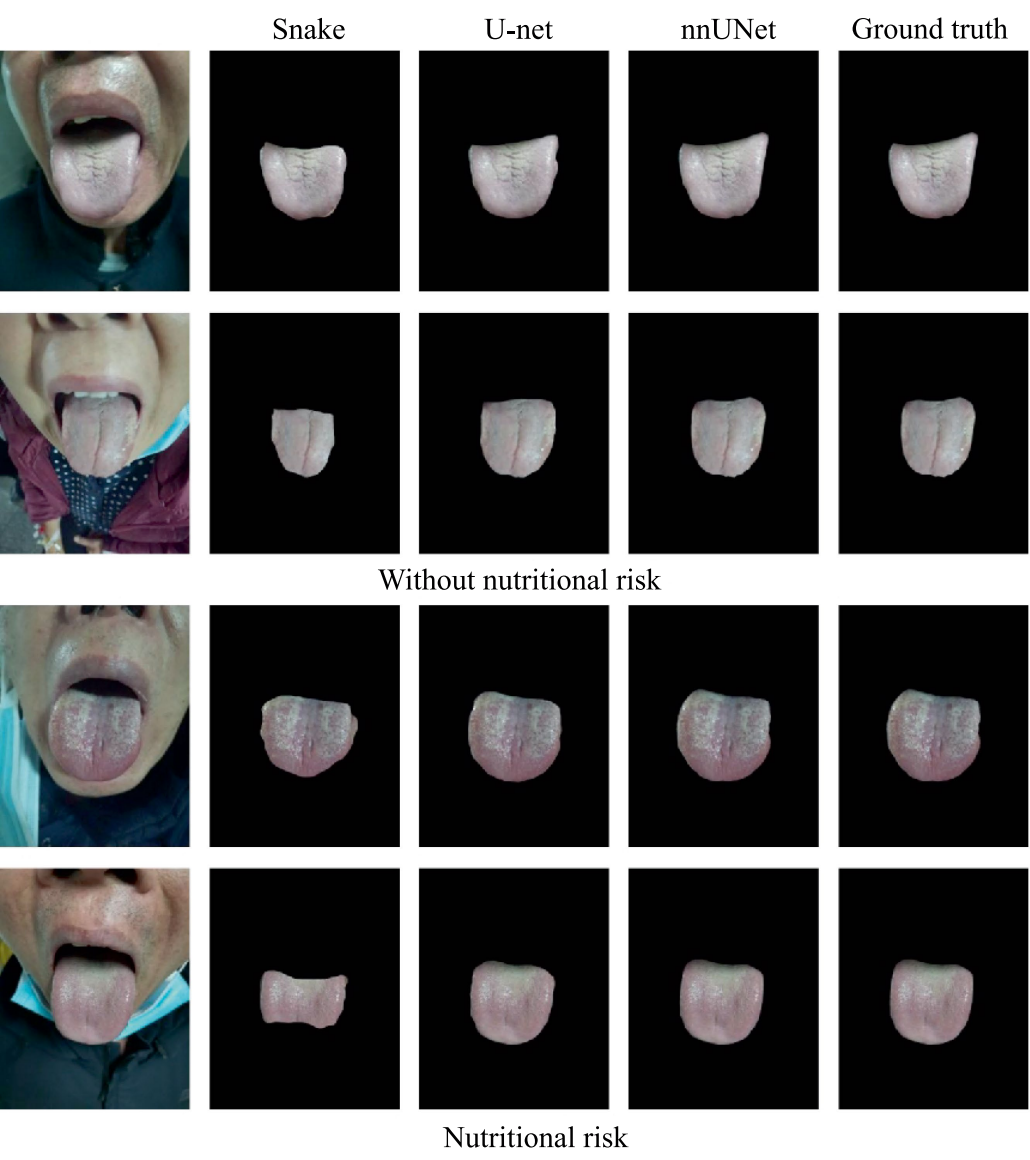
Tongue image dataset distribution and comparison of different segmentation methods.

In this study, patients admitted to the Radiotherapy Department of Qilu Hospital of Shandong University from March to June 2023 were prospectively selected based on the following inclusion criteria: (1) age 18–90 years, (2) NRS2002 nutritional risk screening required (10), (3) hospitalization for more than 24 h, (4) conscious, and (5) enrolment in a disease group with clinical diagnosis of at least one of the following disease types: colorectal cancer (11), lung cancer, esophageal cancer (12), and head & neck tumors. Patients were excluded based on the following criteria: (1) those not eligible for NRS2002 nutritional risk screening, (2) those with serious underlying medical conditions, specifically severe heart failure (NYHA Class III–IV), decompensated liver cirrhosis (Child-Pugh Class C), chronic kidney disease (CKD Stage 4–5), or severe mental illness precluding cooperation, (3) pregnant or breastfeeding women, (4) those unable or unwilling to provide informed consent, and (5) other circumstances considered unsuitable for participation. According to NRS2002 nutritional risk screening results, the participants were divided into at-risk and not-at-risk groups, and representative examples of classification are shown in Figure 2.

It was assumed that the prevalence of nutritional risk in tumor patients was 0.5 and sample sensitivity was 0.8. We performed sample size calculations to ensure statistical validity. Based on a 95% confidence level and 10% confidence interval width, the sample size required for the calculation was determined to be 385 cases. To further improve the accuracy and reliability of the results, we recruited 470 patients, which exceeded the minimum sample size required for calculation and ensured the robustness of the results. The detailed baseline characteristics of the study population, stratified by tumor type, along with the specific comorbidity exclusion standards, are summarized in Supplementary Table S1. With this sample size, we were able to effectively assess the incidence of nutritional risk and associated factors in cancer patients.

Tongue images were acquired using smartphones equipped with high-resolution rear cameras. To ensure data consistency and reproducibility, images were captured under controlled indoor lighting to avoid color distortion. A standardized shooting protocol was strictly implemented: the camera was positioned at a fixed distance of 20–30 cm from the participant’s face and held at a 90° angle perpendicular to the tongue surface. After swallowing, the participants were required to hold the front 1/2 to 2/3 part of the tongue forward and relax, ensuring that the tip of the tongue was down and the surface was flat. A total of two to three composite images were obtained for each participant. The image size was uniformly set to 1,000 × 1,333 pixels, and images were specifically assigned to a training or test set. To ensure data quality, we implemented strict quality control standards, and images that did not meet specific requirements were excluded from the final analysis, for example some images were blurred, had parts of the tongue missing, or included difficult-to-identify coating due to excessive humidity or low resolution.

For the nutritional risk classification task, the preprocessed dataset was partitioned into an 85% training set and a 15% independent test set. To ensure the robustness of the predictive analysis system and mitigate overfitting, a 5-fold cross-validation strategy was implemented during the training phase.

### 2.3 Tongue image segmentation

Tongue image segmentation is an important aspect of linguistics (44). Owing to the presence of distinct facial backgrounds in images, the tongue must be accurately identified and separated to reduce the impact of irrelevant backgrounds on accurate classification. The accuracy of tongue image segmentation directly affects the accuracy of disease diagnosis and recognition. Several automatic language recognition techniques have been widely used, and the U-Net model has demonstrated good performance in various medical image segmentation tasks (5). The improved version, nnUNet, realizes automatic adjustment of the learning rate decay strategy and addresses the balance problem, exhibiting better results (13).

Labelme (version: v5.2.1) was used in this study to manually annotate 470 tongue images in the dataset to outline the outer lines of the tongue. The labeled tongue images were randomly divided into training and test sets at a 4: 1 ratio. The Adam optimizer was used for model training, with beta1 set to 0.9 and beta2 set to 0.99. The input image size was 512 × 512, batch size was set to 32, and epoch was set to 50. The learning rate was initially set to 1e−4 and reduced using the CosineAnnealingLR method to improve model stability. The model was initialized by loading pretraining parameters on the Carvana dataset to estimate the model’s convergence speed and improve the accuracy.

The segmentation performance of Snake, U-Net and nnUNet were evaluated on the verification set in terms of sensitivity, accuracy, dice score, and F1 score. The parameters of the model with the optimal performance on the verification set were selected and applied to the segments of the remaining images in the dataset. nnUNet achieved a sensitivity of 0.9989, Dice score of 0.9979, and F1 score of 0.99986. These results fully demonstrate the effectiveness of our tongue image prediction model in accurately separating tongue images from original images. Different segmentation strategies are also shown on Figure 2.

### 2.4 Comprehensive imbalance processing strategies

To solve the challenges faced by unknown sample datasets in learning and classification, oversampling and undersampling are often adopted at the data level to obtain the best training effect by increasing minority class samples and reducing the majority class samples (6). The tongue image data used in this study showed a significant imbalance in the number of samples between categories (39). Therefore, it was necessary to adopt oversampling and undersampling (14) to improve the sample distribution in the datasets. However, the above operations may lead to model overfitting because only the imbalance between two labels is considered and the sample distribution within the same category is ignored. To systematically address these challenges of dataset imbalance and label ambiguity, we proposed the CLES strategy. First, regarding the majority class, we strategically employed Boundary Undersampling rather than broad cleaning methods such as the Neighborhood Cleaning Rule (NCR). This choice was strictly motivated by the high inter-class similarity inherent in tongue images; unlike NCR which primarily targets noise, Boundary Undersampling specifically removes majority samples located in ambiguous overlapping regions, thereby effectively sharpening the decision boundary to reduce confusion. Second, for the minority class, we implemented a Smooth Oversampling module to generate high-quality synthetic data. Specifically, this module generates new samples via linear interpolation between a minority sample $x_{i}$ and its k -nearest neighbors, as formulated in Equation 1:


$$x_{\mathrm{new}}=x_{i}+\lambda \;(x_{\text{neighbor}}-x_{i})$$ (1)

**Figure 3:**
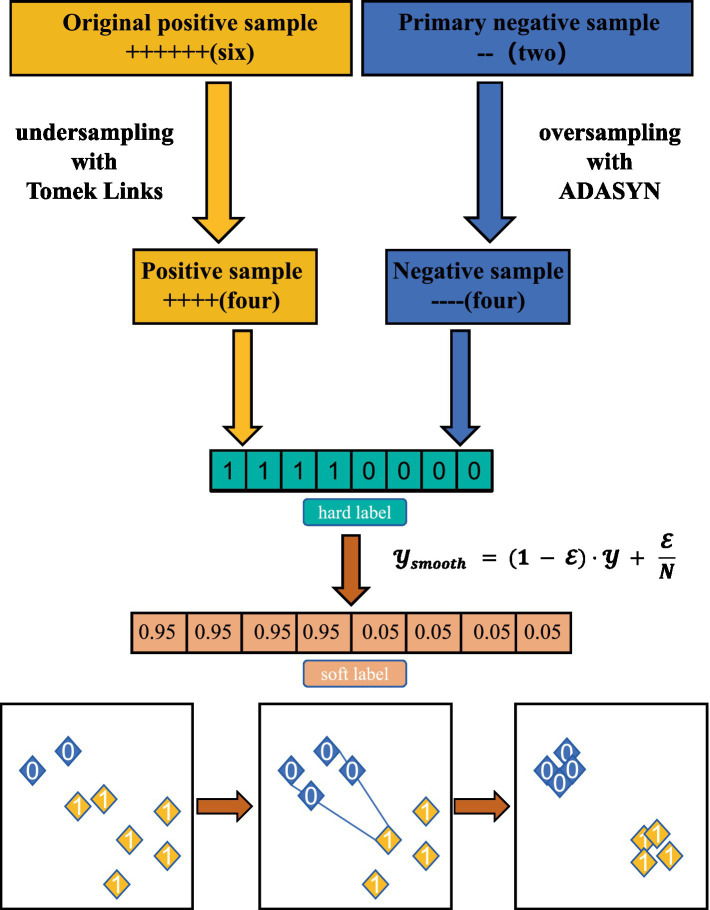
Schematic of the CLES strategy.

We designate this as Smooth Oversampling because the interpolation mechanism fills the sparse regions of the minority class (35), creating a smoother and more continuous feature manifold structure compared to simple replication. Finally, these balanced data are trained using Label Smoothing to mitigate model overconfidence. By synergizing oversampling and undersampling with the label smoothing algorithm, the data distribution within and between classes is balanced, effectively reducing the risk of overfitting. The comprehensive processing strategy of the CLES method is shown in Figure 3.

### 2.5 NRS2002 nutritional risk classification model based on feature SelectNet

In TCM theory, the color, texture, and other features of the tongue can directly reflect the nutritional health of the human body. The extracted features of the tongue were considered as manual features. To avoid brightness interference and obtain tongue image information with more dimensions, the features were mapped to the RGB, YCrCb, and Lab color spaces for color correction. For a large number of extracted features, selecting features with strong correlation enhances the diagnosis accuracy. Influenced by multiple attention mechanisms in the Transformer architecture (15), we designed a feature selection network based on attention mechanisms. Each selection was based on the original input data and information input in the previous step. Consequently, the model selects the relevant attributes from input features for classification.

In the manual feature extraction (16) process, images were mapped into the RGB, YCrCb, and Lab color spaces, and features such as color difference were extracted for each color channel. Using statistics, the grayscale co-incidence matrix was obtained, and indices such as the second-order moment and entropy were obtained to represent the texture features of the tongue images (43).

After extraction, features in the multi-color space were spliced and used as input to the feature selection network model. To reduce the impact of the differences between feature types, the data were normalized before being input into model, and the formula for normalization is given in Equation 2:


$$k\prime =\frac{k-k_{\min}}{k_{\max}-k_{\min}}$$ (2)

where k_max and k_min represent the maximum and minimum values for each data category in all datasets, respectively, k represents raw data, k^’represents normalized data, and the normalization step (17) maps all the data to the [0, 1] interval.

**Figure 4:**
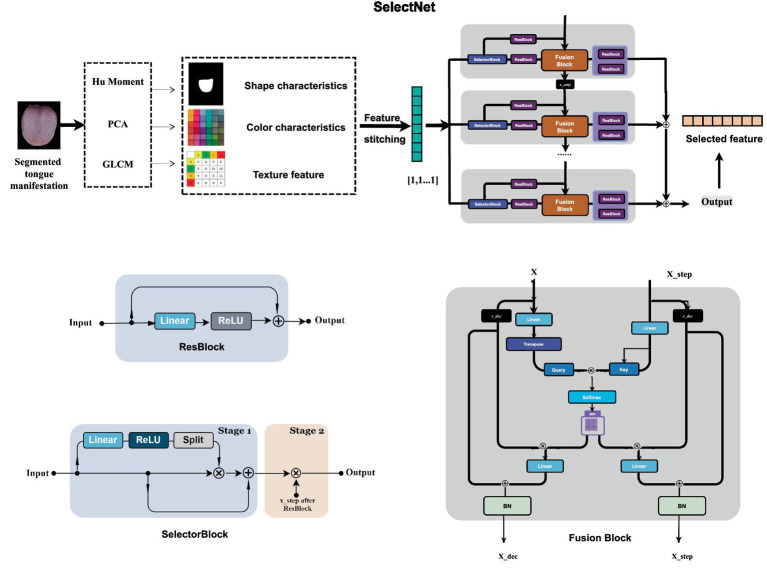
SelectNet framework.

As shown in Figure 4, we designed a deep learning network model for feature selection (18) and fusion, comprising multiple repeated steps similar to the Transformer architecture, each containing a ResBlock, a Choiceblock, and an attention block. The normalized tongue feature was used as the input for the model.

In each step, ResBlock was used to extract and transform the features. In this module, the input *x* first passes through a convolution layer and a ReLU activation function, and then passes through a convolution layer again, and finally the processed features are added to the original input x to obtain the final output, which is connected through the convolution operation, activation function, and residual connection. Extracting higher dimensional features and introducing nonlinearity addresses the problem of network gradient disappearance associated with deep networks. The governing formula for the module is given in Equation 3:


Output=ReLU(Conv(ReLU(Conv(x))))+x (3)

After ResBlock processing, problems such as network gradient disappearance are eliminated with the introduction of a residual jump connection. Then, features are input into the corresponding attention fusion module. After the module generates the corresponding output x_step^‘, which is the input for the next step of the module, x_out is obtained as the final result and selection decision to achieve screening.

For Choiceblock, feature selection depends on the input provided to the model and information transmitted since the last execution, allowing the model to focus fully on the relevant properties of the input features. Inspired by TabNet’s ability to improve model performance through feature selection, Choiceblock comprises two stages, namely Stage 1 and Stage 2: each stage can perform feature selection and transformation operations. In Stage 1, input features are first transformed through a linear layer. Then, ReLU is introduced to bring in nonlinearity, and a split operation is performed to split the features into different parts and generate a set of selection matrices that can be regarded as a weighting process for the input features. In Stage 2, the weighted output feature is multiplied by elements after ResBlock processing, to generate the selection matrix. This matrix is based on the output of the previous step and fused with the output feature of Stage 1 in the form of a product that combines the information of different steps to effectively improve the expression ability of the feature. The two-step design achieves better understanding and utilization of the feature data and improves the prediction accuracy and robustness of the model. The selection matrix is given in Equations 4, 5:


$$S_{1}=\text{Split}(\text{ReLU}(\text{Linear}(\text{Input})))$$ (4)

and


$$S_{2}=\text{Residualblock}\;(x_{\text{step}})$$ (5)

Similar to the Transformer architecture (19), the attention block can realize information fusion and weight allocation of different information, enhance attention to important features of the model by learning the relationship between input features, and fully learn the contextual correlation between multi-level features to further locate and process key information accurately. In addition, multi-scale information is fused; the input feature *x* and the input feature in the previous step are transformed through the linear layer; and the corresponding weight query, key, and value (hereafter referred to as *q*, *k*, *v*, respectively) are generated using softmax to calculate the attention weight, reflecting the importance of different features. Then, the calculated attention weight is multiplied with v to obtain the weighted feature representing fusion $v_{\text{decision}}$ . The input feature is directly matched with the output feature by residual connection to alleviate gradient disappearance, and the output stability of the model is ensured by batch normalization. Weighted feature $v_{\text{decision}}$ , decision output $x_{\text{decision}}$ , and transmission in the next step $x_{\text{step}}^{'}$ are expressed in Equations 6–8:


$$v_{\text{decision}}=\text{Attention}\;(q,k)\cdot v$$ (6)

$$x_{\text{decision}}=\mathrm{BN}\;(x+v_{\text{decision}})$$ (7)

$$x_{\text{step}}^{'}=\mathrm{BN}(x_{\text{step}}+\text{Linear}(v_{\text{step}}))$$ (8)

Following this process, the feature information of the current and previous steps can be fused, and the training process can be stabilized by residual connection and batch normalization, thereby better focusing on regional features and optimizing the fusion effect.

Constructing a classifier for analyzing tongue image features is a key step in constructing a diagnosis model, which directly affects the classification performance of the tongue diagnosis model. In this study, we introduced a decision fusion model constructed by the stacking method. The first-level basic learner of this model includes SVM, RF, KNN, and XGBoost, while logistic regression serves as the first-level meta-learner. By combining the classification results of each base learner, we obtained a more robust learner with stronger generalization ability. Based on the principle of decision fuzziness, feature selection was combined with the construction of the integrated classifier to optimize the modeling process for machine learning models. Note that the constructed NRS2002 nutritional risk prediction model is a machine learning-based classification model. Combining feature selection with the construction of decision fusion classifiers and selecting the optimal learning conditions for each sub classifier can improve the classification accuracy of the model and enhance the overall modeling effect. This comprehensive strategy is essential to improve the accuracy of the model and optimize the modeling results.

### 2.6 Deep spatial location feature prediction model based on the fusion attention mechanism

**Figure 5:**
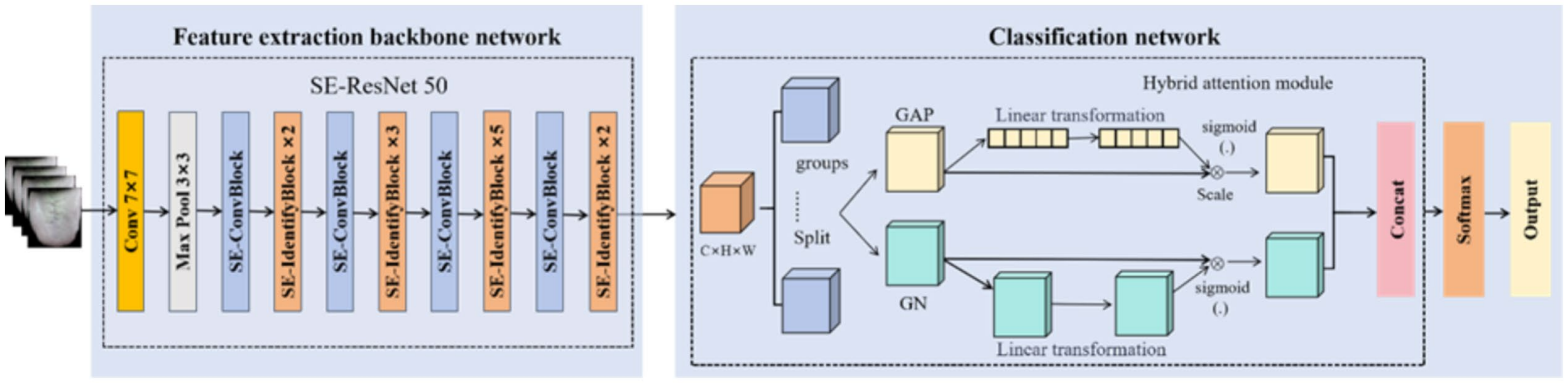
Shuttle attention mechanism classification model.

According to TCM theory, the central region of the tongue image is the “window” that reflects the nutritional status of the human body. Therefore, if the model can pay more attention to the central region during classification training, more relevant location features can be obtained. Moreover, different locations of the central region of the tongue image are closely connected. More spatial feature information can be obtained from the tongue image by studying and comparing the features of different positions. Therefore, we used the ResNet50 with an integrated attention mechanism to extract and classify higher-order depth features of images. We integrated spatial and channel attention mechanisms to enhance feature extraction and improve the quantitative classification of the model. In feature extraction and classification, tongue depth features can be paid higher attention to and accurate quantitative evaluation can be achieved. The framework is shown in Figure 5.

In contrast to manual feature extraction and the general mixed attention mechanism, shuttle attention combines the channel and spatial attention mechanisms (20), paying attention to the local and global depth information of images and their correlations. It is also a lightweight and efficient attention module, leveraging the forward propagation process and global average pooling. The feature map of each channel is compressed into a numerical value to obtain a fixed-size feature vector. We joined the ResBet50 network. This module groups inputs according to channels. For each group of sub-features, shuttle units were used to construct the channel and spatial attention mechanisms.

The implementation process includes the addition of the shuttleblock to the original ResBlock and diving the input features into *n* different groups *X*, as formulated in Equation 9; each group is a sub-feature including one channel. Each sub-feature is further subdivided to generate two corresponding features, each containing one channel, which enter the channel attention branch and the space attention branch, respectively.


$$X=\{X_{1},X_{2},X_{3},\ldots \ldots ,X_{g}\},X_{k}\in R^{\frac{C}{g}\times H\times W}$$ (9)

Here, the channel attention branch does not use the SE (1, 34) module but achieves lightweight design and adopts single-layer linear transformation. Specifically, this part extracts the two sub-features through global average pooling and then performs linear transformation through the fully connected layer and maps the feature vector to the interval (0, 1) using a Sigmoid function. The output weight of the activation function and the other part of the feature map are multiplied by the element to achieve feature fusion and reweighing to highlight the more important location feature information and obtain the channel attention diagram $\;\text{Attentio}n_{c}(X_{k})$ , as formulated in Equation 10.


$$\text{Attentio}n_{c}(X_{k})=\sigma \;(\text{Linear}\;(\mathrm{GAP}(X_{k})))$$ (10)

The spatial attention branch (40) uses group normalization to complete the spatial information statistics of the input feature map and obtains spatial weights and feature fusion following steps similar to the channel attention to highlight the more important spatial feature information and obtain the spatial attention map $\;\text{Attentio}n_{s}(X_{k})$ , as formulated in Equation 11.


$$\text{Attentio}n_{s}(X_{k})=\sigma \;(\text{Linear}\;(\text{Conv}(X_{k})))$$ (11)

Finally, the results of the two attention mechanisms are fused through concatenation to obtain the final fusion feature X′, as illustrated in Equation 12.


$$X\prime =\text{Channel Shuffle}(\text{Concat}(\mathrm{Att}n_{c}{\left(X_{k}\right)}^{{}^{\circ}}\;X_{k},\mathrm{Att}n_{c}{\left(X_{k}\right)}^{{}^{\circ}}\;X_{k}))$$ (12)

To further clarify the structural innovation of the Shuttle Attention (SA) mechanism and distinguish it from existing methods such as CBAM, several key architectural differences are noted. First, unlike CBAM which applies channel and spatial attention sequentially, SA adopts a parallel interaction paradigm via sub-feature grouping. This allows for simultaneous feature recalibration while maintaining a lower computational footprint. Second, the channel branch in SA utilizes a single-layer linear transformation instead of the heavy MLP structure found in SE or CBAM modules, significantly reducing parameter redundancy.

In terms of quantitative efficiency, Shuttle Attention (SA) is engineered for high reliability. By leveraging the Channel Shuffle operation, it facilitates cross-group information flow without the addition of extra convolutional layers, thereby achieving a superior accuracy-latency trade-off. Such task-specific lightweight mechanisms are paramount in medical imaging. Since target organs like the tongue occupy relatively fixed spatial regions, this localized approach effectively mitigates the “over-computation” common in general-purpose global attention models (21). Furthermore, compared to coordinate-based encoding methods such as Coordinate Attention (CA), SA’s emphasis on local spatial information exchange preserves fine-grained tongue textures, including fissures and prickles. These critical diagnostic features are often smoothed out by the 1D global encoding utilized in conventional attention frameworks (45). Given the aforementioned advantages in structural efficiency, computational cost, and local feature preservation, the Shuttle Attention (SA) mechanism demonstrates superior performance in balancing diagnostic accuracy with the requirements for real-time clinical application.

### 2.7 Classification network

The output attention features obtained in the above step are classified into the fully connected layer, which realizes the classification of nutritional risks by learning the direct mapping relationship between feature vectors and category labels and converts them into probability distributions through the softmax function. Finally, the category with the highest probability is selected as the classification result.

By fusing the branch of feature selection information with the attention branch, two scores based on image information and spatial location information can be obtained. Owing to their different emphases, we believe that they have different weights in diagnosis. Therefore, we considered these two prediction models as two features and constructed a fused logistic regression model for fusion. Finally, the accurate NRS2002 nutritional risk prediction model was obtained.

## 3 Results

### 3.1 Calculation results of information fusion model

**Figure 6:**
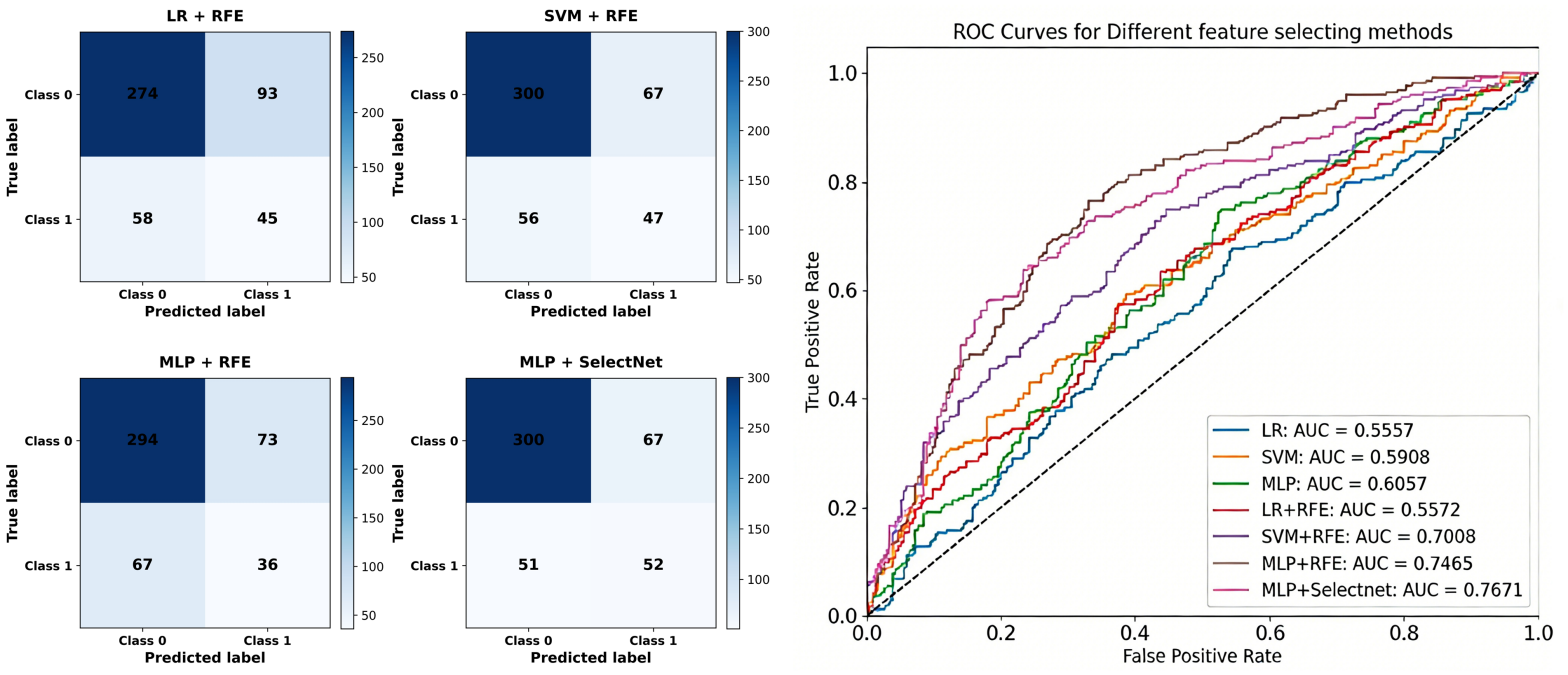
ROC curve and confusion matrix of different feature selection methods.

In this study, a feature selection network was used to predict nutritional risk based on NRS2002, and image features were used to reflect the objective characteristics between tongue image and nutritional risk. However, when a scientific screening method cannot be adopted for a large number of image features, excessive features not only interfere with the prediction but also lead to unsatisfactory model fitting owing to a large amount of computation. Through feature selection, indicators related to nutritional risk can be screened for classification. We demonstrate the necessity of feature selection and the impact of different feature selection methods on training effects on different models. The manual feature classification parameters for different models and feature selection are shown in Table 1, and a comparison between the ROC curve and confusion matrix is shown in Figure 6.

**Table 1:** Test results of different feature selection methods.

Training method	AUC	ACC	Precision	Recall
LR	0.5557	0.5170	0.2958	0.3840
SVM	0.5908	0.6032	0.4163	0.4377
MLP	0.6057	0.5972	0.4221	0.4438
LR + RFE	0.6572	0.6179	0.4958	0.5090
SVM + RFE	0.7008	0.7161	0.4840	0.5570
MLP + RFE	0.7465	0.7282	0.6209	0.6138
MLP+SelectNet	**0.7671**	**0.7904**	**0.8183**	**0.8072**

Bold values indicate the best performance across each metric.

The MLP model (22) combined with the SelectNet feature selection network performed well in tongue image depth feature classification. SelectNet improves the performance of the model by learning the relationships between features and automatically selecting the features that are most useful for classification tasks. Compared to traditional machine learning methods such as LR, SVM, and MLP, MLP + SelectNet showed significant improvements across all the evaluation metrics, indicating that feature selection plays a key role in improving model performance.

In addition, MLP + SelectNet significantly improved performance compared to models that combine recursive feature elimination methods (41, 42), such as LR + RFE, SVM + RFE, and MLP + RFE. Therefore, SelectNet has higher efficiency and accuracy in feature selection and can more accurately identify the most useful features for classification tasks. The use of feature selection network improves model performance significantly, and the MLP classifier using the traditional RFE (23) feature selection achieved the following results: AUC = 0.7565, ACC = 0.7282, Recall = 0.6138, and Precision = 0.6209. When we used our innovative SelectNet for feature selection, ACC, Precision, and Recall improved significantly. Note that SelectNet increased Precision significantly by 19.74%, and the Recall value also showed a significant increase of 19.34%. These results indicate that the model’s screening rate on positive samples was significantly improved, and more features related to nutritional risks could be captured.

In summary, SelectNet performs well in tongue image depth feature classification tasks, and its performance is superior to that of other traditional machine learning and feature selection methods combined with RFE. The results show that SelectNet has significant advantages in improving model performance and provides an effective solution for tongue image feature classification. Through ablation experiments, we demonstrate the effectiveness of the feature selection network. After attention fusion processing by the network, information fusion and weight allocation of different information can be realized. Finally, the connection between various modules can be realized through weighting, and multi-level type feature information can be fused to achieve accurate selection. This underscores the potential of the proposed feature selection network in achieving better feature screening and completing the nutritional risk prediction of NRS2002.

### 3.2 Calculation results of feature balance comprehensive processing strategy

Considering the discrepancy between the positive and negative sample distribution in our dataset and the actual risk distribution in real-world scenarios, we propose a comprehensive processing strategy, CLES, to enhance practical applicability. Given that risk labels often represent interval values (e.g., 0–2 and >3) rather than rigid discrete points, we combined the label smoothing method with the oversampling and undersampling methods. This approach can reasonably smooth the labels while balancing the class distribution and mitigate the risk of model overfitting caused by unbalanced sampling. To better evaluate the effect of CLES treatment strategies, we designed a comparison and ablation experiment. Table 2 shows the results of a comparison between CLES treatment strategies and other unbalanced strategies.

**Table 2:** Test results of different data balancing methods.

Method	ACC	AUC	Precision	Recall
Initial	0.8121	0.8432	0.8067	0.8102
ADASYN	0.8532	0.9002	0.8551	0.8594
Borderline-SMOTE	0.8789	0.9032	0.8554	0.8657
Tomek links	0.8421	0.9006	0.8433	0.8521
CLES	**0.9006**	**0.9198**	**0.8883**	**0.9106**

Bold values indicate the best performance achieved by the CLES strategy.

After applying the unbalanced processing algorithm, the AUC value, ACC value, Precision, and Recall improved significantly. This enhancement indicates that the classification performance is significantly improved when a small number of samples are prioritized. The Recall value reflects the performance of the classification task for positive samples under an unbalanced sampling state. After using CLES, the overall model exhibited significant improvement compared with other unbalanced algorithms, which proves that it is more beneficial to improve the classification performance when paying attention to a few samples and adding label smoothing (24) to prevent the model from overfitting.

Table 3 presents the effectiveness of different deep networks and attention mechanisms for NRS2002 nutritional risk classification. Compared with machine learning methods, the overall performance of deep network classification is improved. ResNet50 had the best classification effect. The ACC value, AUC value, *p* value, and Recall exhibited good results, among which the highest AUC value was 0.9004 and the Recall index was 0.8588. To better demonstrate the effect of the shuttle attention mechanism proposed in this study, we selected the best model, i.e., ResNet50, and added different attention mechanisms to observe how the indicators change. The performance indicators of different attention mechanisms are shown in the lower part of Table 3. Models using different attention mechanisms significantly improved compared to the original model, among which, the shuttle attention mechanism achieved a significantly higher performance than that of other attention mechanisms; the ACC, AUC, and recall rate were significantly higher than those of other attention mechanisms, exceeding 0.900. Compared with the performance indicators of the original model, ACC increased by 3.89% and AUC increased by 1.94%. Precision increased by 7.36% and recall rate increased by 5.18%, which indicates that the aliasing attention mechanism (25) we introduced can extract depth information at different levels by weighting and better focus on information within tongue image regions and feature information between regions. This is consistent with the tongue image in TCM theory, which further demonstrates the theoretical content of tongue image position information reflecting different body conditions in TCM tongue diagnosis. By using the shuttle attention mechanism, the model can pay more attention to the feature relationships between and within regions and better improve the classification accuracy of the model.

**Table 3:** Test results of different feature selection methods.

Training method	AUC	ACC	Precision	Recall
AlexNet	0.7436	0.7833	0.7133	0.7655
VGGNet	0.7802	0.8236	0.7369	0.7834
VIT	0.8102	0.8426	0.7522	0.8286
ResNet50	**0.8617**	**0.9004**	**0.8147**	**0.8588**
ResNet50 + SE	0.8733	0.9084	0.8474	0.8937
ResNet50 + SGE	0.8651	0.9039	0.8256	0.8964
ResNet50 + Shuttle Attention	**0.9006**	**0.9198**	**0.8883**	**0.9106**

Bold values in the ResNet50 row indicate the best performance among the baseline deep networks (without attention); Bold values in the ResNet50 + Shuttle Attention row indicate the overall best performance across all models.

### 3.3 Decision fusion result

In view of the results obtained from the two branches, logistic regression was used to combine the two prediction results. The machine learning method tends to obtain the original manual features of the tongue image, and the model has strong interpretability. The attention mechanism part (26) tends to obtain the depth features and location-related information implied in the tongue image. The results obtained after fusion are shown in Table 4. The results were as follows: AUC = 0.9124, ACC = 0.9270, Precision = 0.8762, and recall rate of 0.9132. Therefore, our framework can predict the nutritional risk of NRS2002 in patients. It also provides a more convenient, fast, and accurate prediction method to achieve clinical early screening and disease prevention.

**Table 4:** Results of fusion strategy.

Method	AUC	ACC	Precision	Recall
Machine method	0.7671	0.7904	0.8583	0.8672
Attention method	0.9006	0.9198	0.8883	0.9106
Mixed method	**0.9124**	**0.9270**	**0.8762**	**0.9132**

Bold values indicate the best performance achieved by the mixed method.

## 4 Discussion

### 4.1 Model explainability analysis

**Figure 7:**
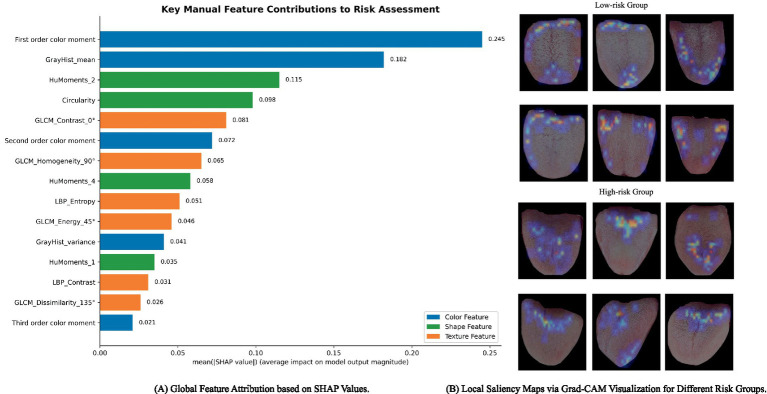
Model explainability analysis.

To bridge the gap between black-box model performance and clinical decision-making (38), we implemented a multi-level interpretability framework combining SHAP-based feature attribution and Grad-CAM spatial visualization, as illustrated in Figure 7.

Specifically, at the global level, we utilized SHAP to quantify the contributions of 107 manual features within the XGBoost classifier. The results in Figure 7A indicate that color-related descriptors, such as the first-order color moment and grayscale mean, are the primary drivers for nutritional risk classification. This provides objective statistical evidence supporting the Traditional Chinese Medicine (TCM) theory that tongue color and coating texture serve as foundational biomarkers for assessing a patient’s metabolic and nutritional status.

At the local level, Grad-CAM saliency maps provide spatial validation of the model’s focus. The visualization in Figure 7B reveals a distinctive “centralized attention” pattern in the high-risk group, with the model’s focus densely localized on the central and posterior regions of the tongue. According to TCM diagnostics, the central tongue region is a critical “window” reflecting the functional state of the spleen and stomach, which are directly linked to nutritional absorption. The high consistency between the model’s localized attention and these canonical diagnostic zones demonstrates that the Shuttle Attention mechanism successfully prioritizes clinically relevant pathological irregularities while effectively filtering out irrelevant background noise.

Finally, to enhance the credibility for clinical application, we empirically confirmed the effectiveness of these key features through the superior performance achieved via 5-fold cross-validation on the 85% training set and evaluation on the independent 15% test set. These high-accuracy classification results demonstrate that the color and spatial features captured by the model possess not only statistical significance but also clear clinical value in pathological assessment. This validation paradigm ensures that our framework provides high-precision quantitative assessment while maintaining semantic transparency aligned with medical logic.

### 4.2 Limitations and future perspectives

Although NRS2002 has been widely used owing to its high efficiency compared with other questionnaires (27), it has inherent limitations in two key aspects: the dependence of patients on self-reported weight change and difference in the professional level of evaluators (33), and the operational efficiency limits the dynamic detection. TCM tongue diagnosis is non-invasive and has the potential for rapid nutritional assessment to support the global needs of primary healthcare systems because tongue features are associated with gastrointestinal pathology and nutritional status (36). However, its subjective interpretation and poor reproducibility hinder its clinical application. Emerging AI technology, specifically the analysis of tongue images using deep learning, can address these gaps. Previous studies focused on image preprocessing or color correction but lacked integration with NRS2002 correlation (37).

To address the lack of standardization and weak correlation with pathology in traditional tongue image analysis in nutritional risk assessment, this study proposed a dual-channel model combining low-dimensional manual features with CNN deep features (28) and combined them with attention mechanisms to locate key diagnostic areas. Through the double branch architecture of machine learning (29) and deep learning, the model not only retains the interpretability of manual features (such as tongue color and texture) guided by TCM theories but also uses a CNN to extract deep spatial features such as tongue coating thickness. At the same time, a feature selection network is introduced to optimize and screen a large number of manual features. Combined with the “shuttle attention” mechanism, the spatial position and regional internal features are captured synchronously to form complementary feature expressions. The standardized preprocessing process based on nnUNet effectively solves interference problems, such as uneven illumination and tongue shape change, and significantly improves the robustness of feature extraction.

In this cohort study involving cancer patients, the model showed excellent performance in the NRS2002 nutritional risk stratification task, with an AUC of 0.9124 and ACC of 0.9240. These results are a significant improvement compared to those of the single-feature model. By establishing a quantitative mapping between the tongue phenotype and NRS2002 threshold, this study revealed the relationship between specific tongue characteristics and nutritional risk and provided an objective basis for TCM syndrome differentiation. In the dynamic monitoring scenario, the screening time was shortened from 5 min to real-time detection, and there was no need to rely on auxiliary clinical indicators. This solves the problems of cumbersome operations and poor dynamics of traditional methods.

This study has the following limitations. First, the subject population was limited to Chinese patients, and the inclusion of multi-ethnic/racial groups (30) in future studies will help enhance the cross-racial applicability of the model. Second, the current prediction model based on tongue image characteristics (31) of patients with colorectal cancer, esophageal cancer, lung cancer, and head and neck tumors needs to be further enriched with sample data of other diseases to improve the model’s identification capability and clinical promotion value. Third, the sample size needs to be further expanded, and key technical parameters such as standardization and resolution of tongue image acquisition equipment need to be improved. Finally, the model performance needs to be continuously validated and optimized through prospective clinical studies to establish a dynamic updating mechanism to ensure its clinical practicability.

## 5 Conclusion

This study constructed an innovative NRS2002 nutritional risk intelligent prediction model based on multi-modal tongue image features. Based on the deep feature fusion architecture (32) of image-location information and combined with the collaborative optimization of the multi-level feature analysis module and dynamic classification network, the hierarchical prediction efficiency of nutritional risk was significantly improved. This study not only validates the scientific value of the TCM tongue diagnosis theory but also promotes the evolution of tongue image analysis technology to a standardized and non-invasive direction. The mobile terminal interactive system developed through the research and development realizes the automatic processing of the entire “shooting, analysis and report” process through a lightweight architecture. The standardized feature analysis framework established by this technology not only provides an objective and quantitative dynamic monitoring tool for clinical practice but also highlights its interpretable interaction design more effectively and supports the optimization of nutrition intervention decision-making, thereby providing a technical basis for the construction of an evidence-based medical insurance evaluation system.

## Data Availability

The original contributions presented in the study are included in the article/Supplementary material, further inquiries can be directed to the corresponding authors.
