# Correction: Tongue image analysis for accurate prediction of nutritional risk screening in cancer patients during radiochemotherapy: a feature selection network and aliasing attention mechanism approach

**DOI:** 10.3389/fnut.2026.1846974

**Published:** 2026-04-24

**Authors:** Bowen Yang, Aohan Li, Abdul Haleem Mohib, Xu Qiao, HuaiDong Li, Cong Wang, Chang Liu, Henan Zhang, Yukun Zhang, Shuying Li, Shanghui Guan, Shasha Zhao

**Affiliations:** 1 Center of Intelligent Medicine, School of Control Science and Engineering, Shandong University, Jinan, Shandong, China; 2 Department of Radiation Oncology, Shanghai Pulmonary Hospital, Tongji University School of Medicine, Shanghai, China; 3 Department of Hospitality and Business Management, Technological and Higher Education Institute of Hong Kong, Hong Kong, China; 4 Department of Radiation Oncology, Qilu Hospital, Shandong University, Jinan, Shandong, China; 5 Department of Nutrition, Qilu Hospital, Shandong University, Jinan, Shandong, China

**Keywords:** attention mechanism, computer aided diagnosis, NRS2002, nutriology, tongue image diagnosis, tumornutrition analysis

In the published article, there was an error in the affiliation(s) for the following authors: Henan Zhang, Yukun Zhang, Shuying Li, and Shanghui Guan.

The incorrect affiliation for these authors was given as:

^2^ Department of Radiation Oncology, Shanghai Pulmonary Hospital, Tongji University School of Medicine, Shanghai, China

The correct affiliation for these authors is:

^4^ Department of Radiation Oncology, Qilu Hospital, Shandong University, Jinan, Shandong, China

The original version of this article has been updated.

