# Supplementary material for: Tongue image analysis for accurate prediction of nutritional risk screening in cancer patients during radiochemotherapy: a feature selection network and aliasing attention mechanism approach
Source: Front Nutr. 2026 Jan 12;12:1752250. doi: 10.3389/fnut.2025.1752250 (PMC12833964; doi:10.3389/fnut.2025.1752250)
Supplement: Supplementary file 1 [file Table_1.pdf]

Supplementary Table S1: Baseline characteristics of the study and comorbidity exclusion standards.

Variable	Total (N=470)	Lung Cancer (n=240)	Colorectal Cancer (n=75)	Esophageal Cancer (n=72)	Head & Neck Tumor (n=44)	P-value
**Age (years)**	60.4 ± 12.7	59.9 ± 14.7	59.6 ± 9.3	65.2 ± 9.9	55.6 ± 11.4	**0.001**
**Gender**						**<0.001**
Male	336 (71.5%)	179 (74.3%)	40 (53.3%)	60 (83.3%)	37 (84.1%)	
Female	135 (28.5%)	62 (25.7%)	35 (46.7%)	12 (16.7%)	7 (15.9%)	
**NRS2002 Score**						**<0.001**
Score 1	251 (53.4%)	136 (56.4%)	45 (60.0%)	28 (38.9%)	21 (47.7%)	
Score 2	115 (24.5%)	60 (24.9%)	15 (20.0%)	19 (26.4%)	13 (29.5%)	
Score ≥ 3	104 (22.1%)	45 (18.8%)	15 (20.0%)	25 (34.7%)	10(22.7%)	
**Nutritional Risk**						**0.038**
At-risk (NRS ≥ 3)	104 (22.1%)	45 (18.8%)	15 (20.0%)	25 (34.7%)	10 (22.7%)	
Non-risk (NRS < 3)	366 (77.9%)	196 (81.3%)	60 (80.0%)	47 (65.3%)	34 (77.3%)	

Note: Data are presented as mean ± SD or n (%). P-values were calculated using ANOVA for age and Chi-square test for categorical variables. Bold values indicate statistical significance ($p < 0.05$).

Exclusion Criteria: Patients were excluded if they were ineligible for NRS2002, pregnant/breastfeeding, unable to consent, presented other unsuitable circumstances, or had serious underlying medical conditions. Specific severity criteria for the latter included: (1) Heart Failure: NYHA Class III–IV; (2) Liver Disease: Decompensated cirrhosis (Child-Pugh Class C); (3) Kidney Disease: CKD Stage 4–5; (4) Severe mental illness precluding cooperation.
